# The Effect of Weight Loss on the Muscle Proteome in the Damara, Dorper and Australian Merino Ovine Breeds

**DOI:** 10.1371/journal.pone.0146367

**Published:** 2016-02-01

**Authors:** André M. Almeida, Rui G. Palhinhas, Tanya Kilminster, Timothy Scanlon, Sofia van Harten, John Milton, Dominique Blache, Johan Greeff, Chris Oldham, Ana Varela Coelho, Luís Alfaro Cardoso

**Affiliations:** 1 Centro Interdisciplinar de Investigação em Sanidade Animal, Faculdade de Med. Veterinária, Lisboa, Portugal; 2 ITQB/UNL–Instituto de Tecnologia Química e Biológica, Universidade Nova de Lisboa, Oeiras, Portugal; 3 Ross University School of Veterinary Medicine, PO box 334, Basseterre, St. Kitts and Nevis (West indies); 4 Department of Agriculture and Food Western Australia, Perth, WA, Australia; 5 Institute of Agriculture, University of Western Australia, Perth, WA, Australia; University of Florida, UNITED STATES

## Abstract

Seasonal Weight Loss (SWL) is an important constraint, limiting animal production in the Tropics and the Mediterranean. As a result, the study of physiological and biochemical mechanisms by which domestic animal breeds respond to SWL is important to those interested in animal breeding and the improvement thereof. To that end, the study of the proteome has been instrumental in gathering important information on physiological mechanisms, including those underlying SWL. In spite of that, little information is available concerning physiological mechanisms of SWL in production animals. The objective of this study was to determine differential protein expression in the muscle of three different breeds of sheep, the Australian Merino, the Dorper and the Damara, each showing different levels of tolerance to weight loss (low, medium and high, respectively). Per breed, two experimental groups were established, one labeled “Growth” and the other labeled “Restricted.” After forty-two days of dietary treatment, all animals were euthanized. Muscle samples were then taken. Total protein was extracted from the muscle, then quantified and two-dimensional gel electrophoresis were conducted using 24 cm pH 3–10 immobiline dry strips and colloidal coomassie staining. Gels were analyzed using Samespots^®^ software and spots of interest were in-gel digested with trypsin. The isolated proteins were identified using MALDI-TOF/TOF. Results indicated relevant differences between breeds; several proteins are suggested as putative biomarkers of tolerance to weight loss: Desmin, Troponin T, Phosphoglucomutase and the Histidine Triad nucleotide-binding protein 1. This information is of relevance to and of possible use in selection programs aiming towards ruminant animal production in regions prone to droughts and weight loss.

## Introduction

Seasonal weight loss (SWL) is one of the major constraints to animal production in the tropics and the Mediterranean [1]. The weather of such geographic regions are characterized by seasonal patterns of rainfall, resulting in distinct “dry” and “rainy” seasons. As we have described previously, the available quality nutrition in pastures is very poor during the dry season in South [2–3] and Western Africa [4–5], as well as Western Australia [6]. Farmers living in these regions implement supplementation strategies to counteract the effects of SWL. While effective, supplementation as a means to an end is both costly and difficult to implement [7].

Alternatively, and as a response to the prohibiting cost of supplementation, there is the potential use of breeds that have evolved in harsh climates that are, as a result, more tolerant of SWL. The selection of appropriate livestock breeds aims for SWL tolerance that improves the productivity of both quantitative and qualitative traits. The study of metabolic responses of different breeds to SWL can contribute to an improved understanding of the genomic and physiological changes caused by SWL. Studies thus far have largely been conducted at the genomic level. Post-genomic tools, particularly proteomics, have played an increasingly major role in animal selection as well as other areas of animal and veterinary sciences [8–9]. Although working with farm animal proteomics poses some challenges and limitations [10], the technique has been implemented with important results in meat science [11–12], mainly in species of cattle [13], rabbit [14] and pig [15]. More recently, significant work has been accomplished with proteomics concerning sheep species (*Ovis aries*) through an in-depth characterization of the proteome of ovine muscle longissimus lumborum [16].

The effects of SWL on several physiological parameters in ruminants, particularly the lipid [17] and Nitrogen [18–19] metabolisms concerning productive [2] and reproductive [3] traits have been studied at length. Recent trends focus on the effects of SWL on protein expression profiles; of note is the comparison of different breeds of domestic animals and their differing levels of adaptation to nutritional stress. The comparison of Wild Iberian and New Zealand white rabbits is one such example with each species demonstrating important differences in structural proteins and glycolytic pathway enzymes of the gastrocnemius muscle [20].

In this study, we examined the effects of experimentally induced weight loss on the proteomic profiles of the ovine gastrocnemius muscle. We used three breeds with differing levels of adaptation to nutritional stress: the Damara, the Dorper and the Australian Merion. The Damara is a fat tailed breed originating from Southern Africa (South Angola and Namibia) that evolved in areas bordering the Kalahari Desert; it is still bred by two tribes local to the area, the *Himba* and *Herero* [21]. In recent years, it has been exported to other regions of the world [21]. Damaras, like most fat-tailed sheep breeds, are well adapted to the stresses of SWL [21] and have the ability to digest poor quality fodders [22]. It has recently been established that the Damara has a particular lipid metabolism that leads to the accumulation of branched chain fatty acids in its tail, muscle and liver, an uncommon feature in ruminants [23]. The Dorper is a breed that was developed in South Africa, a result of crosses between Blackhead Persians and Dorset Horns. It is classified as a breed with an intermediate tolerance to SWL [21]. Finally, the Merino is a well-known wool producing breed that is considered to be more susceptible to seasonal weight loss than the other two breeds. We have previously characterized the productive performance [6], carcass traits [24] and muscle fatty acid composition of these three breeds [25]. We have also characterized the gene expression of the regulatory enzymes of the intermediate metabolism in the liver [26]. More recently a shotgun proteomic analysis of wool proteins in relation to feed restriction was conducted on the same Merino animals involved in this study [27].

In this study we used two-dimensional electrophoresis (2DE) coupled to protein identification by mass spectrometry to explore breed differences in proteomic profiles of gastrocnemius muscle. Our main objective is to suggest potential biomarkers of tolerance to SWL.

## Material and Methods

### Animals and Experimental Design

The animal trial was conducted at the Merredin Research Station (Merredin, WA, Australia), using experimental design and nutritional treatments that were previously described [6]. Seventy-two, six month old ram lambs comprised of Merino, Dorper and Damara breeds were randomly assigned to one of two diets. They were then separated based on breed into groups with twelve animals per experimental group: Merino Restricted (MeR), Merino Growth (MeG), Damara Restricted (DaR); Damara Growth (DaG), Dorper Restricted (DoR) and Dorper Growth (DoG). Animals were purchased from registered breeders and were considered to be representative of each breed. All animals were exclusively fed on the same commercial roughage-based feed pellet (Macco 101, Macco Feeds, Williams, WA, Australia), with 9.3 MJ/kg dry matter (DM) of metabolized energy (ME), 115 g/kg DM of crude protein (CP) and 291 g/kg of acid detergent fiber (ADF). The fatty acid content of the feed pellet was 15.1 g/kg DM, and the major fatty acids (in percentage of total fatty acids) were 18:1 cis-9 (32.5%), 16:0 (30.0%), 18:2 n-6 (27.9%), 18:0 (4.3%) and 18:3 n-3 (2.1%) [23]. All animals had free access to drinking water. Nutritional treatments were individually calculated using the Freer equation [28] as previously described [6]. Using this approach, animals in the restricted feed groups would lose weight at approximately 100g/day (85% of maintenance), whereas animals in the growth group would gain approximately 100 g/day. Animals were weighed twice a week in order to record growth performance. The experiment lasted 42 days. By the end of this period, restricted fed animals had a percentage of weight loss of 12–14%, whereas animals that gained weight had increases in the range of 7–13%; for further details, please refer to [6]. At the end of the trial, animals were slaughtered in a commercial abattoir (Tammin abattoir, Tammin, WA, Australia), following commercial practices [24]. Immediately after the skinning and evisceration processes, samples from the gastrocnemius muscle were excised, snap frozen and kept at -80°C until further analysis.

### Insulin and leptin quantifications and statistical analysis

Blood was collected from the jugular vein for each animal on days 0, 21 and 42, before feeding into heparinized tubes. Blood was immediately centrifuged, then harvested for plasma. Plasma was kept at -20°C until further analysis. Plasma concentrations of insulin and leptin were quantified in duplicate for all samples using double antibody radioimmunoassay methods previously described [28]. The intra-assay coefficients of variability were less than 7% for both assays at any concentration. Statistical differences among groups were determined using a 3 x 2 factorial (3 breeds in 2 feeding conditions) analysis of variance using STATISTICA (StatSoft, Inc., 2004, version 7, Tulsa, OK, USA). Whenever a significant difference at P<0.05 was detected a post-hoc comparison test (Tukey test) was performed.

### Protein extraction and Quantification

From the 72 samples, four samples per experimental group were randomly chosen and used for protein extraction and quantification. Muscle proteins were extracted using the protocol previously described for rabbit gastrocnemius muscle [20]. Forty to fifty milligrams of frozen muscle sample was removed with a scalpel blade and homogenized in 1ml of extraction buffer 1: 8.3 M Urea, 2 M Thiurea, 1% (w/v) DTT (Dithiothreitol), 2% (w/v) CHAPS (3-[(3-Cholamidopropyl)dimethylammonio]-1-propanesulfonate hydrate) with an Ultraturrax T8 homogenizer (IKA Werke, Staufe, Germany), followed by 30 minutes vortex in a cold-room at 4°C and centrifuged at 10 000×g for 30 minutes. After the centrifugation, supernatant was transferred to a new tube and immediately snap-frozen for later use. Protein concentrations were determined using the 2D Quant kit (GE Lifesciences, Uppsala, Sweden) following manufacturer’s instructions.

### Two-Dimensional Gel Electrophoresis

Isoelectric focusing (IEF) followed by SDS-PAGE was used to separate proteins. The adequate volumes, with a total of 600 μg of protein per extract, were diluted to 470 μl in 3-[(3-cholamidopropyl)dimethylammonium]-1-propane sulfonate (CHAPS) (2%, w/v), urea (8 M), IPG buffer pH 3–10 (0.5%, v/v) and dithiothreitol (DTT) (0.02 mM), and used for IEF. Electrophoresis was performed according to Almeida et al. [20] with some modifications. An IPGphor system (Amersham Biosciences, Uppsala, Sweden) and immobiline drystrips of 24 cm with a linear pH gradient from 3 to 10 (GE Healthcare, Uppsala, Sweden) were used. The IEF started with 30 V for 12 h followed by 3h at 100 V, 1.5 h at 300 V, 1.5 h at 500 V, 2h at 1000V, a voltage gradient of 3h until 8.000 V and a final step at 8.000 V until reaching 125.000 V/hour.

After IEF, strips were stored at -80°C for later use. Before SDS-PAGE, IEF strips were equilibrated in two steps using 10 ml of Tris-HCl (pH 8.8; 50 mM) with urea (6 M), glycerol (30%, v/v) and SDS (2%, w/v) [20]. Dihiothreitol at 10 mg/ml was used in the first and iodoacetamide at 25 mg/ml in the second equilibration steps. The equilibration step lasted 15 min under slow agitation [20]. After equilibration, the second dimension was conducted using 12.5% polyacrylamide gels on an Ettan Dalt Six electrophoresis system (GE Healthcare, Piscataway, NJ, USA) using the running conditions recommended by the manufacturer (10 mA/gel, 80 V, 1 W/gel during 1 hour followed by 15 W/gel until the dye front reached the end of the gel).

Gels were stained using Colloidal Coomassie Blue G-250 [29]. Gels were stained for 48 h and subsequently washed 3 times in double distilled water. Gels were stored at 4°C in a 20% (w/v) ammonium sulphate solution until image acquiring and band excision. One gel was run per muscle sample per animal in the experimental group.

### Gel Digitalization, Image and Statistical Analysis

Digital images of the gels were acquired in an Imagemaster digital scanner [20]. Gels were analyzed using Progenesis SameSpots software (Nonlinear Dynamics, Newcastle upon Tyne, UK) following manufacturer’s instructions for visible stain gels as recently described [30]. All statistically significant (p<0.05) spots with a power over 0.8 were manually excised, using a disposable scalpel, for digestion and subsequent identification by Matrix-Assisted Laser Desorption Ionization-Time Of Flight/Time Of Flight (MALDI-TOF/TOF).

### Protein Identification

Spots selected were manually excised for individual in-gel digestion with trypsin as described [20]. Spots were washed with 30μL of water for 30 minutes, washed in acetonitrile (50%), reduced with 10mM DTT at 56°C for 45 minutes, alkylated with 55mM iodoacetamide for 30 minutes, washed in acetonitrile (100%) and vacuum dried (SpeedVac^®^, Thermo, Waltham, MA, USA). Gel pieces were rehydrated with a digestion buffer (50 mM NH_4_HCO_3_ buffer) containing trypsin (Promega, Madison, WI, USA) and incubated overnight at 37°C. The digestion buffer containing peptides was acidified with formic acid, desalted and concentrated using C8 microcolumns (POROS R2^®^, Applied Biosystems, Foster City, CA, USA), as described [20].

Protein identification was conducted using a MALDI-TOF–TOF data acquired with an Applied Biosystem 4800 Proteomics Analyzer (Applied Biosystems, Foster City, CA, USA) in both MS and MS/MS mode [31]. Positively charged ions were analyzed in the reflectron mode over the m/z range of 800–3500 Da. Each MS spectrum was obtained in a result independent acquisition mode with a total of 800 laser shots per spectra and a fixed laser intensity of 3500 V, which was externally calibrated using des-Arg-Bradykinin (904.468 Da), angiotensin 1 (1296.685 Da), Glu-Fibrinopeptide B (1570.677 Da), ACTH (1–17) (2093.087 Da), and ACTH (18–39) (2465.199) (Calibration Mix from Applied Biosystems). Fifteen best precursors from each MS spectrum were selected for MS/MS analysis. MS/MS analyses were performed using CID (Collision Induced Dissociation) assisted with air, using a collision energy of 1 kV and a gas pressure of 1 × 106 Torr. Two thousand laser shots were collected for each MS/MS spectrum using a fixed laser intensity of 4500 V. The S/N ratio was set at 20 as recommended by manufacturer. Raw data were generated by the 4000 Series Explorer Software v3.0 RC1 (Applied Biosystems, Foster City, CA, USA) and all contaminant m/z peaks originating from human keratin, trypsin autodigestion, or matrix were included in the exclusion list used to generate the peptide mass list used in the database search.

The generated mass spectra were used to search the Swissprot predicted protein database with a taxonomical restriction setting (mammal database). Searches were conducted using Mowse from MASCOT-demon 2.1.0 Software (Matrix-Science) algorithm. Protein identifications were accepted if the protein score was above a threshold of 95% (p < 0.05). The interpretation of the combined MS + MS/MS data was carried out using the GPS Explorer Software (Version 3.5, Applied Biosystems, Foster City, CA, USA) with the following parameters set: missed-cleavage, one; peptide tolerance, 50 ppm; fragment mass tolerance, 0.25 Da; fixed modification, carbamidomethylation of cysteine; and variable modification, methionine oxidation. From the predicted protein database, the theoretical molecular mass and pI of the identified proteins were obtained using the Expasy Mw/pI Tool (http://www.expasy.org/tools/pi_tool.html). The identified proteins were only considered if a MASCOT protein score above 61 (p < 0.05) was obtained.

### Animal welfare disclaimer

All work involving animals was conducted according to relevant international guidelines (European Union procedures on animal experimentation—Directive 2010/63/EU) that regulate the use of production animals in animal experimentation. These define that in the case of experiments carried out under standard production conditions, no approval from an ethics committee is required. Nevertheless, this experiment was conducted with the approval of the Ethics Committee of the Department of Agriculture and Food Western Australia (DAFWA, Perth, WA, Australia) registered as process 07ME06. The entire trial was conducted under the supervision of the veterinary authority in the State of Western Australia. Author AM Almeida holds a FELASA (Federation of European Laboratory Animal Society Associations) grade C certificate that enables designing and carrying out animal experimentation under European Union regulations. Animal management, handling, transport and slaughter were all conducted replicating approved standard commercial practices in the Commonwealth of Australia and in the State of Western Australia. Animals included in this experiment were therefore subjected to the same welfare conditions as production animals.

## Results

### Live weight changes

The evolution of live weight of the animals used in this experiment has been described [6,24] and is summarized in table 1. Animals on the restricted diet lost 14% (Merino) and 12% (Damara and Dorper) of their initial weight at the onset of the trial. Animals on the growth diet increased by 7%, 10% and 13% for the Damara, Merino and Dorper groups, respectively.

**Table 1:** Live weight changes (kg ± standard deviation) for Damara, Dorper and Merino breeds assigned to either a restricted or growth diet.

	Restricted Feeding Diet			Growth diet		
Day	Merino	Damara	Dorper	Merino	Damara	Dorper
0	32.90±4.9	42.00±7.0	37.90±6.0	34.20±4.4	43.10±7.4	39.50±7.9
42	28.60±3.1	37.30±5.3	34.30±5.6	37.70±4.5	46.00±5.6	45.00±7.5
Decrease / Increase	-14%	-12%	-12%	+10%	+7%	+13%

Significant differences (P<0.05) were found for all experimental groups in a comparison of day 42 and day 0 and between the two groups of the same breed at day 42. Table adapted from [24]

### Insulin and leptin concentrations

Insulin and leptin concentrations on day 0, 21 and 42 are presented in table 2. There was no effect of the interaction between diet and breed on plasma concentrations of leptin and insulin on any day. On the first day’s trial both leptin and insulin concentrations were influenced by breed (P<0.05), with the Damara sheep presenting higher values compared to Merino and Dorper animals (+52% and 47%, respectively). Additionally, for day 21 and 42, higher leptin levels (respectively 28% and 29% higher than in Merino and 17% and 24% higher than in Dorper) and higher insulin levels (respectively 63% and 66% higher than in Merino and 52% and 71% higher than in Dorper) in Damara animals were observed. All control animals revealed higher concentrations of both leptin and insulin compared to animals on restricted diets on days 21 and 42.

**Table 2:** Plasma concentrations of leptin and insulin (mean (s.e.)) in three breeds of sheep fed a diet maintaining or restricting growth.

		Merino		Damara		Dorper		Significance level		
	Day	Growth	Restricted	Growth	Restricted	Growth	Restricted	Breed	Diet	BxD
Leptin (ng/ml)	0	0.501 (0.015)	0.541 (0.018)	0.773 (0.084)	0.810 (0.088)	0.547 (0.032)	0.533 (0.023)	***(1)	NS	NS
	21	0.953 (0.046)	0.706 (0.067)	1.208 (0.134)	0.919 (0.084)	1.118 (0.090)	0.698 (0.040)	*(2)	***	NS
	42	0.883 (0.057)	0.638 (0.049)	1.084 (0.126)	0.873 (0.104)	0.951 (0.055)	0.627 (0.048)	*(1)	***	NS
Insulin (μU/ml)	0	3.671 (0.469)	2.703 (0.271)	7.632 (1.259)	5.828 (0.981)	3.463 (0.580)	2.815 (0.278)	***(1)	NS	NS
	21	4.636 (0.453)	3.049 (0.275)	7.552 (0.977)	4.938 (0.576)	5.014 (0.430)	3.221 (0.309)	***(1)	***	NS
	42	4.223 (0.470)	2.322 (0.232)	6.938 (0.821)	3.958 (0.468)	4.318 (0.348)	2.067 (0.111)	***(1)	***	NS

Trial duration– 42 days; n = 72; Restricted feeding level– 85% of maintenance. Data is shown as averages. (1)—Da≠ M; Da≠Do; M = Do; (2)—D≠M; Da = Do; M = Do;

*—p<0.05

***—p<0.001.

NS = Non significant; BxD—Interaction effect of breed and diet

### Two-Dimensional electrophoresis

**Fig 1:**
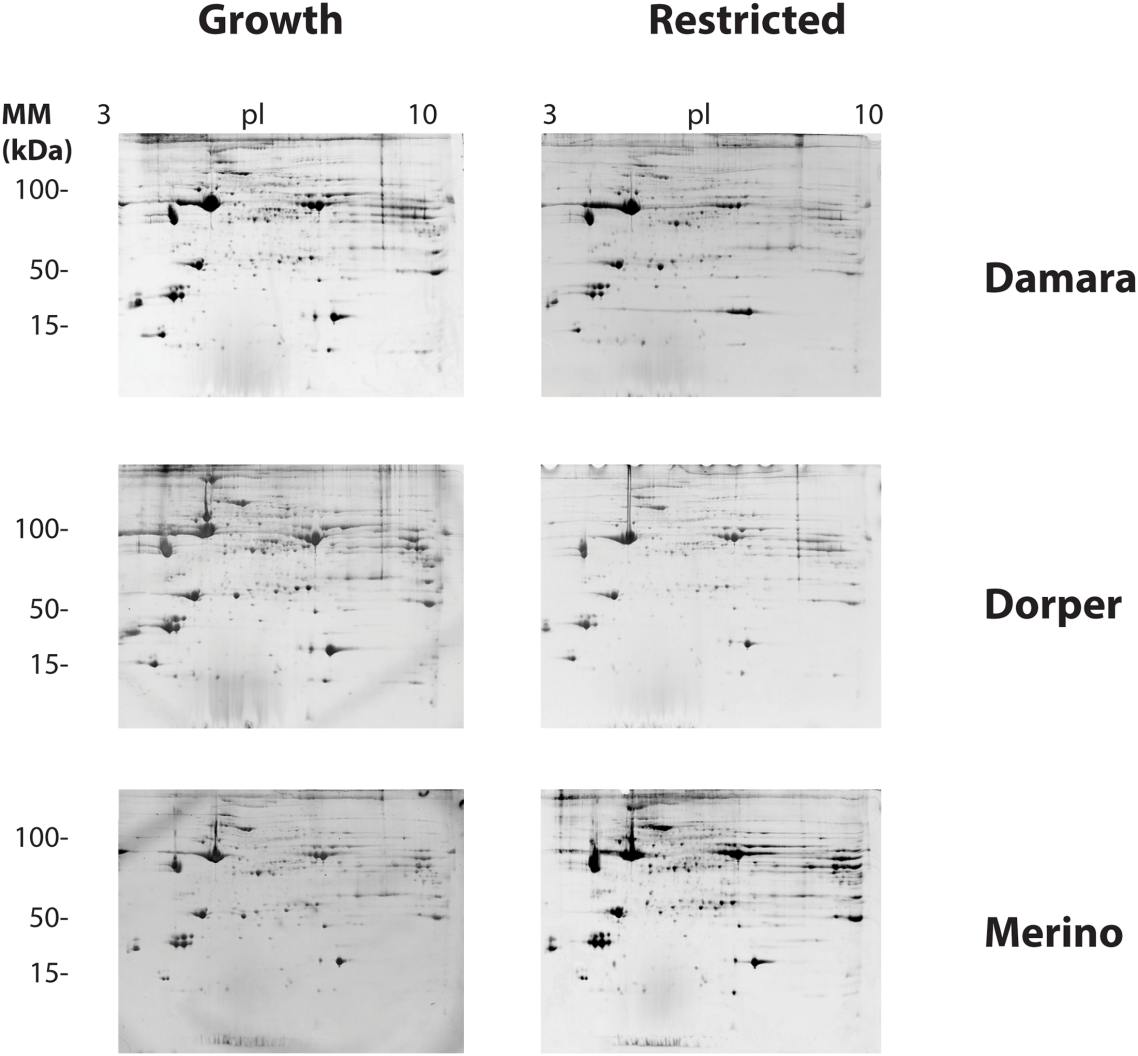
Two-dimensional gel electrophoresis. Examples of 2D gels obtained for the muscle tissue of Merino, Damara and Dorper rams. Molecular mass markers (MM, vertical scale), as well as isoelectric point (horizontal scale) are also presented. Two-Dimensional electrophoresis was carried out in linear 24cm Immobiline dry strips (pH 3–10). Six hundred micrograms of protein were loaded per gel. Staining was performed with Colloidal Coomassie Blue G-250.

Analogous protein amounts were loaded per gel and treatment. Accordingly, the electrophoresis was considered successful as we have achieved an adequate separation of proteins according to pI and molecular mass. Representatives of the two-dimensional electrophoresis gels produced in this study are shown in Fig 1.

### Protein expression analysis and identification

**Fig 2:**
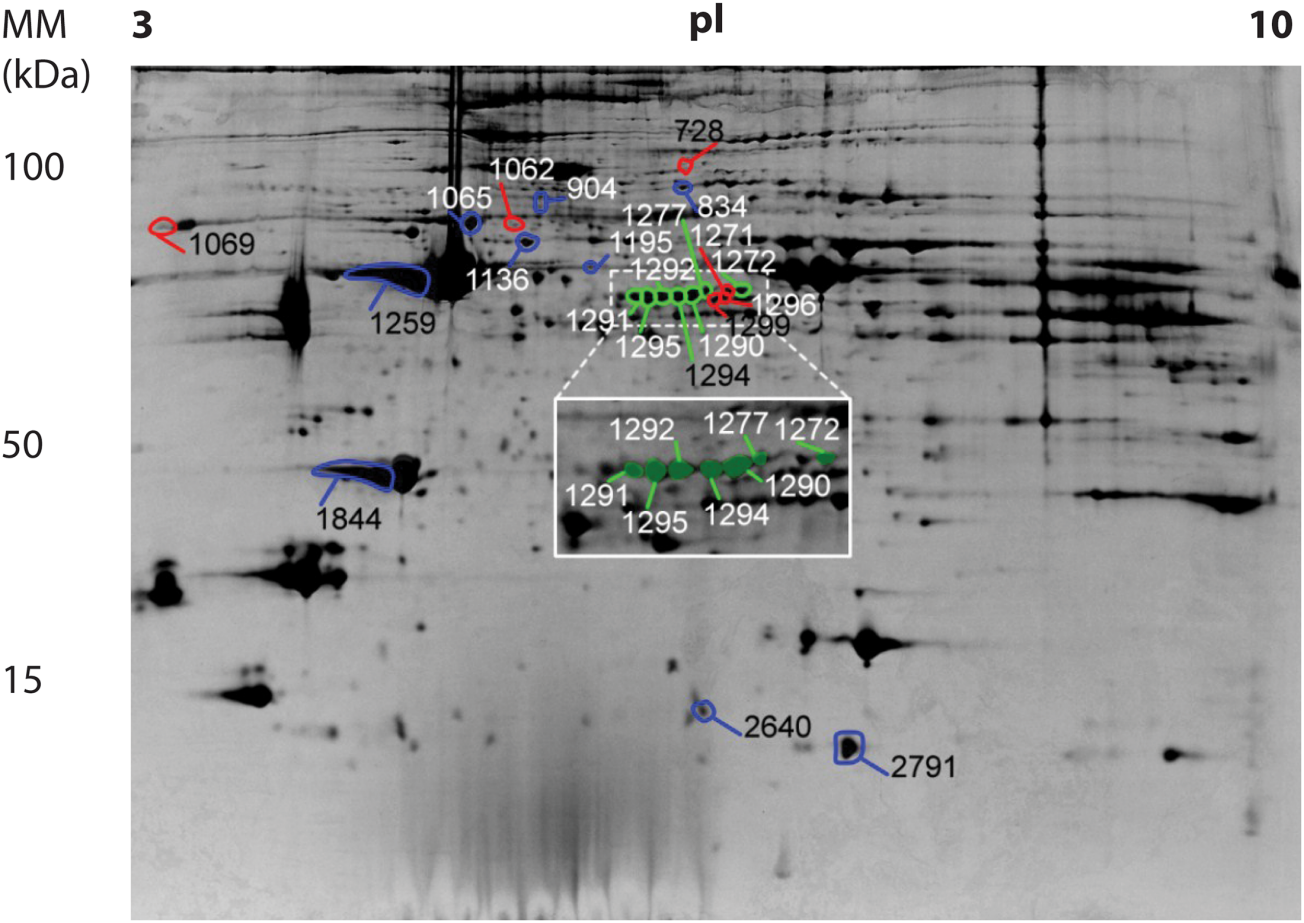
Two-dimensional gel electrophoresis. Example of a 2D gel for a merino ram gastrocnemius muscle, showing the spots that were selected for identification. Molecular mass markers (MM, vertical scale), as well as the isoelectric point (pI, horizontal scale are also presented. Two-dimensional electrophoresis was carried out in linear 24 cm Immobiline DryStrips (pH 3–10). Gels were stained with colloidal Coomassie Blue G-250.

Gel analysis for the muscle tissue revealed a total of 22 spots where differential expression between at least two of the experimental groups was observed. Such proteins are identified in a representative gel in Fig 2. Of these, we were able to identify a total of 16 proteins. Expression results are presented in table 3. Tables include a summary of the identification parameters.

**Table 3:** Protein spots showing differential expression in at least 2 experimental groups (p<0.05).

Spot reference	Protein name	Merino		Damara		Dorper		Accession Number	Matched Peptides^a^		Sequence Coverage (%)^b^	Protein Score^c^
		Growth	Restricted	Growth	Restricted	Growth	Restricted		MS	MS/ MS		
834	Phosphoglucomutase -1	1.579e+004	1.968e+004	1.038e+004	1.143e+004	1.586e+004	1.526e+004	PGM1_BOVIN	16	1	30	286
904	T complex protein 1 subunit theta	1.530e+004	1.858e+004	1.348e+004	1.132e+004	1.211e+004	0.972e+004	TCPQ_BOVIN	7	1	14	92
1065	Desmin	10,70e+004	10.34e+004	6.370e+004	9.448e+004	8.330e+004	5.314e+004	DESM_BOVIN	23	10	53	688
1136	Cytochrome b-c1 complex subunit 1, mitochondrial	5.213e+004	5.170e+004	6.425e+004	4.590e+004	3.931e+004	3.473e+004	QCR1_BOVIN	14	7	34	452
1195	Actin, alpha skeletal muscle	1.092e+004	1.226e+004	2.171e+004	2.376e+004	2.449e+004	1.598e+004	ACTS_BOVIN	6	1	23	154
1259	Actin, alpha skeletal muscle	8.763e+005	12.20e+005	9.940e+005	12.32e+005	7.631e+005	9.543e+005	ACTS_BOVIN	11	8	42	419
1272	Troponin T, fast skeletal muscle	3.807e+004	1.891e+004	4.608e+004	3.711e+004	3.159e+004	2.867e+004	TNNT3_BOVIN	7	3	23	262
1277	Troponin T, fast skeletal muscle	1.720e+004	1.210e+004	2.170e+004	1.704e+004	1.409e+004	1.793e+004	TNNT3_BOVIN	3	1	14	72
1290	Troponin T, fast skeletal muscle	9.358e+004	4.829e+004	11.71e+004	8.938e+004	7.157e+004	6.769e+004	TNNT3_BOVIN	7	3	24	262
1291	Troponin T, fast skeletal muscle	2.346e+004	1.533e+004	2.453e+004	1.981e+004	2.954e+004	3.049e+004	TNNT3_BOVIN	7	1	21	183
1292	Troponin T, fast skeletal muscle	3.679e+004	2.714e+004	5.420e+004	4.380e+004	4.052e+004	4.609e+004	TNNT3_BOVIN	8	1	24	173
1294	Troponin T, fast skeletal muscle	5.089e+004	3.553e+004	5.338e+004	3.372e+004	6.681e+004	6.491e+004	TNNT3_BOVIN	6	1	21	196
1295	Troponin T, fast skeletal muscle	5.142e+004	3.255e+004	6.358e+004	4.785e+004	5.329e+004	5.663e+004	TNNT3_BOVIN	3	1	11	61
1844	Myosin light chain 1/3, skeletal muscle isoform	3.380e+005	4.402e+005	2.885e+005	4.771e+005	2.867e+005	3.347e+005	MYL1_BOVIN	8	5	50	582
2640	Histidine Triad nucleotide-binding protein 1	2.143e+004	2.110e+004	1.471e+004	1.228e+004	2.618e+004	1.957e+004	HINT1_BOVIN	1	1	11	87
2791	Hemoglobin subunit beta	0.570e+005	1.294e+005	1.063e+005	0.969e+005	1.538e+005	1.695e+005	HBB_SHEEP	8	4	77	467

^a^Number of peptides, matching the identified protein, whose sequence differs in at least one amino acid residue;

^b^Percentage of the identified protein sequence covered by the matched peptide

^c^Identification Score obtained with the Mowse algorithm. A result is considered to be significant when a score above 61 is attained.

## Discussion

Seasonal Weight Loss (SWL) in ruminants is a pressing issue with significant impact on animal production, particularly in the Mediterranean and tropics. Food restriction and SWL have significant effects on animal metabolism and many breeds differ in their response to SWL [1]. Understanding the mechanisms by which such breeds are able to tolerate SWL is paramount to animal selection. Its successful application to the metabolism of domestic animals makes proteomics a valuable tool for efficiently establishing molecular biomarkers of tolerance to SWL in other species [9]. Such biomarkers can in turn be used in selection programs, particularly those targeting the development of higher-productive breeds or types of breeds able to tolerate SWL. In order to begin answering questions about breed suitability, we adopted an approach comparing three different sheep breeds with different levels of tolerance to SWL, the Damara, the Dorper and the Australian Merino.

### Plasma concentrations of insulin and leptin

For every collection event, Damara sheep had significantly higher plasma concentrations of leptin and insulin when compared to the other two breeds, with the exception of leptin on day 21, which was similar in Damara and Dorper animals. The absence of difference in leptin concentrations between the Damara and Dorper on Day 21 could be contributed to the fact that both breeds have a higher rate of fat deposition than that of theMerino [24]. The higher levels of both insulin and leptin in the Damara on day 0 and day 42 compared to the other two breeds could be explained by the fact that Damara are a member of the fat-tailed sheep, which are known to accumulate body fat in their tail. That store can contribute up to 25.95% of the total body fat of the animal [32]. Plasma leptin levels are highly correlated with body fat [33] and the fat in the tail of the Damra is composed of adipocytes, which do not exist in the other two breeds, potentially explaining why the Damara produces more leptin then the Dorper or Merino. The contribution of the fat tail in leptin secretion, however, is not supported by results recently presented [34]; leptin concentrations were not found to be significantly different between thin-tailed and fat-tailed sheep. The idea that the tail is responsible for differences in leptin concentrations is, thus, merely a hypothesis.

The higher insulin levels measured in the Damara and Dorper could explain the higher body fat % reported in each breed [24]. Insulin is responsible for stimulating lipogenesis and fatty acid esterification, while simultaneously inhibiting fatty acid oxidation, causing higher body fat storage in ruminants. Both the high concentration of insulin, which stimulates the expression of leptin in ruminants, and the high level of body fat that produces more leptin, could be responsible for the higher leptin levels found in the Damara and Dorper [34].

Plasma leptin and insulin were decreased in animals exposed to feed restriction during 21 or 42 days. These results are in agreement with previous studies [35–36] and [37] and are possibly due to the decrease of stimulation of leptin expression by insulin during feed restriction [36].

### Proteome analysis: structural and contractile apparatus proteins

In the present study, we have identified three structural and contractile apparatus proteins showing different expression levels: desmin (1 spot), α actin (2 spots), Myosin Light Chain (MLC, 1 spot) and finally seven spots that were identified as Troponin T. Not all proteins showed a similar pattern. In fact, where actin and MLC seemed to increase as a consequence of food restriction within each individual breed, for troponin T the opposite was noted. In the case of these three proteins, similar results were attained for the control groups in the three breeds. For desmin, yet another pattern was observed with both the experimental and control Merino groups having very similar results; there was an increase in desmin with SWL for the Damara (1.5 fold) and a 60% decrease as a consequence of SWL for the Dorper breed. Additionally, considering the control groups of each breed, it must be stressed that Merinos show the highest value, Dorper an intermediate value and Damaras the lowest value of desmin. These results imply that the Damara breed is better adapted to nutritional stress, and not only has a lower expression of desmin overall when compared to the other breeds, but also seems to react to weight loss through an increase in desmin expression. This protein is a muscle-specific type III intermediate filament, essential for proper muscular structure and function. Decreases in their expression have been associated in humans with severe skeletal myopathies [38]. This role coupled with the results obtained in the Damara breed could be interpreted as a tendency for the animal to maintain muscle structure and function when subjected to weight loss despite the muscle tissue mobilization that occurs as a result of feed restriction. This pattern of desmin was not observed in the other two breeds and was unique to the Damara.

Actin has been shown to increase expression levels as a consequence of genotype, with breeds possessing comparatively higher muscular development showing similarly higher expression of actin as opposed breeds with lower muscular development. This has been demonstrated in rabbits [20] and in sheep breeds such as the Texel with muscle hypertrophy phenotypes and by comparison to controls [39]. It has not, however been described in cattle muscle hypertrophy [40]. In this study, results for the control groups of the three breeds are very similar for spot 1259 (a spot with a higher abundance), while in spot 1195 (a spot with a lower abundance) a two fold increase was detected between Dorper, Merino and Dorper and between Damara and Merino. The observed results are in accordance with the link between higher actin expression and muscle deposition, as Dorpers and Damaras are predominantly meat producing animals. Merinos, on the other hand, have been primarily selected for intensive wool production, with meat production being of secondary interest [24]. Regarding the nutritional status, previous results in similar studies with Boer goat bucks [18] and rabbits [20] indicate that actin expression does not change in correlation to nutritional status. In our study, there seems to be an increase in expression with weight loss from 25% (Damara and Dorper, spot 1259) and 40% (Merino, spot 1259). Such results could be explained by an effort in all breeds to maintain muscle structure and functionality in situations of muscle depletion. Our results are contrary to others in the literature such as those described by Kim et al. [41], who demonstrated working with Longissimus dorsi in *Hanwoo* cattle that actin is clearly up-regulated when comparing earlier (12 months) to later (27 months) stages of the fattening process. It is also up-regulated when animals’ growth needs are substantially higher than stages during which animals are depositing fat. Both cases refer to comparisons between two opposing nutritional states and should be taken with caution given the existing differences between weight loss and growth processes.

MLC has been associated with higher muscle deposition ability in rabbits [20] and cattle [40], but not in sheep [39]. When comparing steers at different stages of the fattening process, Kim et al. [41] detected higher expression levels in later stages of the process. Shibata et al. [42] demonstrated that MLC expression was increased in animals fed on pasture as opposed to concentrate. These results are corroborated in our own experiment and point to relevant MLC expression increases as a consequence of nutritional status (1.29, 1.67 and 1.23 fold respectively in Merino, Damara and Dorper sheep). The breed observed to be most tolerant of SWL, the Damara, showed the highest increase in MLC expression of the three as a consequence of weight loss.

Troponin T fast type is another protein with key relevance in muscle structure and contractility. This protein has been found to be over-expressed in grazed cattle when compared to grain fed cattle [42] and to decrease its expression in cattle with a double muscle phenotype [40]. Our previous results with Troponin I in rabbit gastrocnemius muscle consistently indicates an over-expression in under-fed animals [20]. Increased Troponin expression can thus be hypothesized to be associated with lower muscular development and/or with lower planes of nutrition. The results obtained in this experiment (for example, see spot 1290) from animals whose feed is restricted express lower levels of Troponin I than respective controls of the same breed. Additionally, the breed with higher muscle deposition levels, the Dorper, showed lower levels of Troponin expression than those seen in the Merino and Damara animals. These results corroborate the suggestion that Troponin overexpression is a marker of lower muscular development as well as of under-nutrition.

### Proteome analysis: metabolism proteins

We have identified four proteins with a metabolic role that had differential expression levels: Phosphoglucomutase (spot 834), Cytochrome b-c1 complex subunit 1, mitochondrial (spot 1136), T complex protein 1 subunit theta (spot 904) and Histidine triad nucleotide-binding protein 1 (spot 2640). Phosphoglucomutase (PGM) is an enzyme that plays a key role in glycogenolysis and glycogenesis. In humans, its deficiency leads to severe metabolic disorders related to glucose metabolism such as dilated cardiomyopathy and exercise intolerance [43]. In cattle, PGM has recently been associated with meat tenderness [44]. Our results point to higher expression levels of PGM in the breeds that have higher muscle deposition, the Dorper and Merino, whereas Damara sheep display lower levels of expression. Previous studies have shown that PGM is over-expressed during muscle hypertrophy in both cattle and sheep [39–40]. Given its role in glycogenolysis, it would be expected that PGM would increase its expression as a consequence of feed restriction. In our study, this only happens for the Merino breed where a 25% increase was registered, indicating that the other two breeds were not as severely affected as the Merino. It could therefore be suggested that this enzyme serve as a biomarker for weight loss susceptibility.

Cytochrome b-c1 complex subunit 1(UQCRC1) is a mitochondrial protein involved in oxidation/reduction process and plays a major role in the cell’s electron transport and respiratory chain. It has long been described as a biomarker for ageing in the skeletal muscle of female humans [45]. More recently, this protein has also been noted to play a significant role in ovine Corpus Luteum regression [46] and has been shown to increase expression levels in the ventricle proteome of hypertensive rats in response to exercise [47]. It also increases expression in glycolytic muscle of leptin-deficient obese mice, albeit while maintaining expression levels in oxidative muscle [48]. UQCRC1 is therefore strongly associated with various catalytic pathways. Our results show that the Damara breed is more affected by weight loss in conjunction with UQCRC1 expression than the other breeds. The Damara shows a decrease in expression levels by 30%, while the other two breeds show results very similar to levels recorded in control groups. These results could be interpreted as an enhanced ability of the Damara breed to counteract catabolic activity in the gastrocnemius muscle as a consequence of weight loss by decreasing it to levels below their basal expression, a capacity not shared by the other two breeds. Also of note is the fact that Damara sheep show UQCRC1 basal levels higher than those recorded for Dorper and Merino. These results are of particular interest as it has been demonstrated that the expression of this protein is increased in obese animals [48] and the gene itself has been associated with body lipid accumulation in high-intramusclular fat Wagyu X Limousine crosses [49]. The fact that Damara muscle is very lean, lacking intramuscular fat [24,50] coupled with the fact that this breed accumulates lipids within the fat-tail adipose tissue [21] means the resulting distinct lipid metabolism of the Damara [23] may be a major contributor to this expression pattern.

T Complex protein 1 (TCPQ) is a molecular chaperone involved in protein folding, particularly actin and tubulin and, by extension, is involved in cytoskeletal organization [51]. Lower expression levels of TCPQ has been associated with low quality grades of beef as related to intramuscular fat content and meat tenderness [52]. These results are in accordance with ours that show expression levels of TCPQ in the Damara and Dorper are lower than in the Merino, likely because the first two have leaner meats [24] and in the case of the Damara, its afore mentioned distinct lipid metabolism [23]. Undernutrition was also found to increase TCPQ expression levels in the merino but not in the other two breeds. Such results may be interpreted as a consequence of higher muscle depletion levels in the Merino breed as a consequence of weight loss which would lead to alterations in cytoskeletal organization via actin folding. This corroborates the results obtained for actin mentioned in the previous section.

Finally, Histidine Triad nucleotide-binding protein 1 (HINT1), formerly known as Protein Kinase C Inhibitor-1, is a protein that is involved in a number of processes, particularly transcription, translation, inflammation and apoptosis [53]. The protein has been proposed as a marker for chronic, mild stress in the hypothalamus of depressed rats, where it increases its expression in pathological situations characterized by depressed weight gain [54]. Somewhat inversely but in tandem with this oncept, our results point toward a slight decrease as a consequence of weight loss in all breeds, an unsurprising result as transcription and translation would likely decrease as a consequence of the body mobilizing reserves. Also interesting is the fact that expression levels in control groups is lower in the Damara than in the other breeds, possibly explained by lower transcription and translation levels in this breed with less muscular development than the other two.

### Conclusions and Future Perspectives

In this study, we have compared the muscle proteomes of three sheep breeds subjected to weight loss. This is an original approach that, with the exception of results obtained working with New Zealand White and wild Iberian rabbits, has never been conducted before. We compared Australian Merinos with two breeds from Southern Africa, one particularly well adapted to nutritional stress, the Damara, and one considered to have an intermediate level of adaptation, the Dorper. The latter also has the characteristic of being selected for high muscle growth and carcass yield. Results point out several interesting differences between the breeds, particularly at the level of the structural proteins. These characteristics are often associated with different levels of muscle development between the Dorper and the other two breeds and also of a particular lipid metabolism linked to the fat-tailed adipose tissue characteristic of the Damara. With these results, it is also possible to propose several candidates as putative markers of tolerance to weight loss: Desmin, Troponin T, Phosphoglucomutase and the Histidine Triad nucleotide-binding protein 1. These proteins are of potential use in selection programs aimed at improving animal production in regions prone to droughts and weight loss. Going forward, in order to fully comprehend differences between breeds and the SWL adaptation phenomenon, it is important to extend the analysis described here to other tissues of significant metabolic role with particular focus on the liver and adipose tissues.
